# Placement of Triblidiaceae in Rhytismatales and comments on unique ascospore morphologies in Leotiomycetes (Fungi, Ascomycota)

**DOI:** 10.3897/mycokeys.54.35697

**Published:** 2019-06-18

**Authors:** Jason M. Karakehian, Luis Quijada, Gernot Friebes, Joey B. Tanney, Donald H. Pfister

**Affiliations:** 1 Farlow Herbarium of Harvard University, 22 Divinity Avenue, Cambridge, MA, 02138, USA Harvard University Cambridge United States of America; 2 Universalmuseum Joanneum, Centre of Natural History, Botany & Mycology, Weinzöttlstraße 16, 8045 Graz, Austria Centre of Natural History, Botany & Mycology Graz Austria; 3 Pacific Forestry Centre, Canadian Forest Service, Natural Resources Canada, 506 West Burnside Road, Victoria, BC V8Z 1M5, Canada Natural Resources Canada Victoria Canada

**Keywords:** Convergent evolution, desiccation-tolerant fungi, discomycetes, fungal ecology, systematics, spore morphology, taxonomy

## Abstract

Triblidiaceae is a family of uncommonly encountered, non-lichenized discomycetes. A recent classification circumscribed the family to include *Triblidium* (4 spp. and 1 subsp.), *Huangshania* (2 spp.) and *Pseudographis* (2 spp. and 1 var.). The apothecia of these fungi are persistent and drought-tolerant; they possess stromatic, highly melanized covering layers that open and close with fluctuations of humidity. Triblidialean fungi occur primarily on the bark of *Quercus*, Pinaceae and Ericaceae, presumably as saprobes. Though the type species of *Huangshania* is from China, these fungi are mostly known from collections originating from Western Hemisphere temperate and boreal forests. The higher-rank classification of triblidialean fungi has been in flux due in part to an overemphasis on ascospore morphology. Muriform ascospores are observed in species of *Triblidium* and in *Pseudographiselatina*. An intense, dark blue/purple ascospore wall reaction in iodine-based reagents is observed in species of *Pseudographis*. These morphologies have led, in part, to these genera being shuffled among unrelated taxa in Hysteriaceae (Dothideomycetes, Hysteriales) and Graphidaceae (Lecanoromycetes, Ostropales). Triblidiaceae has been placed within the monofamilial order Triblidiales (affinity Lecanoromycetes). Here, we demonstrate with a three-gene phylogenetic approach that triblidialean fungi are related to taxa in Rhytismatales (Leotiomycetes). We synonymize Triblidiales under Rhytismatales and emend Triblidiaceae to include *Triblidium* and *Huangshania*, with *Pseudographis* placed within Rhytismataceae. A history of Triblidiaceae is provided along with a description of the emended family. We discuss how the inclusion of triblidialean fungi in Rhytismatales brings some rarely observed or even unique ascospore morphologies to the order and to Leotiomycetes.

## Introduction

In 2015 J.M.K. made a collection of *Triblidiumcaliciiforme* Rebentisch: Fries during a New Brunswick Museum BiotaNB bioblitz. Our research on this species revealed that it is one of a handful of North American specimens (Magnes 1997; Farr and Rossman 2019; Mushroom Observer 2019; MyCoPortal 2019). Around this time other North American collections were made by J.M.K., J.B.T. and L.Q. of *Pseudographispinicola* (Nylander) Rehm and *T.caliciiforme*. As we undertook our research G.F. made several collections of various species of *Triblidium* Rebentisch: Fries and one of *Pseudographiselatina* (Acharius) Nylander in Austria. Species in these two genera are more extensively represented in European fungaria (Magnes 1997) than in North America. Our collective morphological observations of these specimens in the living state have provided the impetus to undertake this study of the systematics of Triblidiaceae Rehm as emended by Magnes (1997). For some of these species, the molecular characters that we have generated are the first to be used in research.

Magnes’s *Weltmonographie der Triblidiaceae* (1997) is the primary reference for our study. Magnes (1997: 27–28) adopted Eriksson’s (1992) circumscription of Triblidiaceae to include three genera: *Triblidium* (4 spp., 1 subsp.), *Pseudographis* Nylander (2 spp., 1 var.) and *Huangshania* O. E. Eriksson (2 spp.). We employ “triblidialean fungi” as a term of convenience to collectively refer to these genera. Magnes’s concept of the family is as follows.

**Figure 1.**
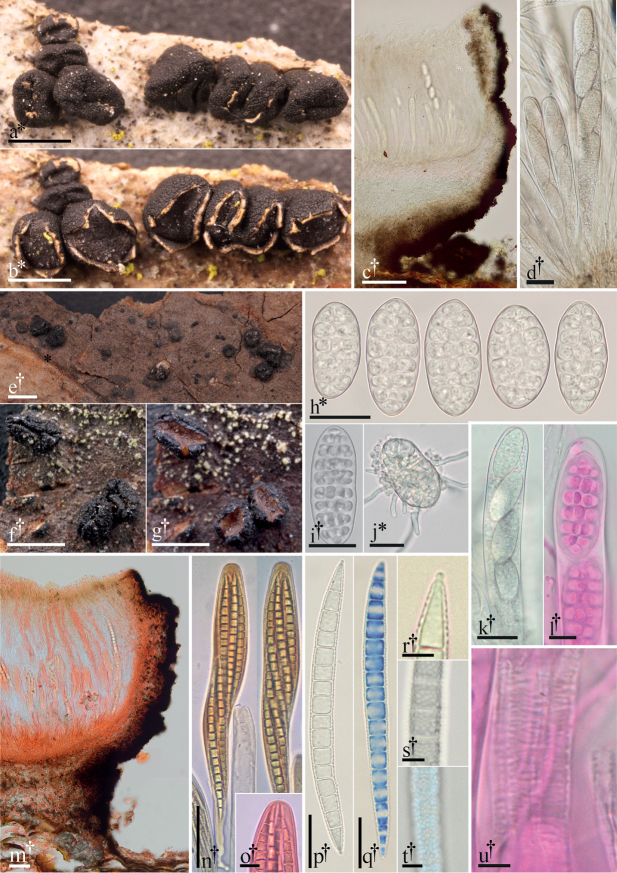
Morphological features of Triblidiaceae. **a–d, h–l, u***Triblidiumcaliciiforme***a** dried apothecia on bark **b** same apothecia hydrated **c** 15 µm thick longitudinal section **d** dead asci containing living ascospores **h–i** ascospores **j** germinating ascospore **k** dead ascus containing living ascospores, detailing the apex **l** ascus detailing the apex (phl) **u** fine transverse striations of dehisced ascus (phl). **e–g, m–t***Huangshaniaverrucosa***e** habit of apothecia on bark (dried) **f** detail of dried apothecia **g** detail of same apothecia hydrated **m** 15 µm thick longitudinal section in (Cr) **n** asci (KOH & Mlz) **o** detail of ascus apex (Cr) **p** ascospore **q** ascospore (cb/l) **r** detail of plug-like structure in a terminal cell of an ascospore **s–t** detail of verrucose ascospore surface (t in cb/l). All microphotographs of cells and tissues mounted in water unless otherwise noted: Congo red (Cr), cotton blue in lactophenol (cb/l), Melzer’s reagent (Mlz), phloxine (phl), potassium hydroxide (KOH). † = dead, * = living. Scale bars: 1 mm (**a–b, e–g**); 50 µm (**c, m–n**); 20 µm (**h–k, p–q**); 10 µm (**l, o**); 5 µm (**r–u**). Specimens photographed: *T.caliciiforme*: **a–b, d, j** GJO-0088904; **i** FH-15071105; **c, h, k–l, u** CUP-18080101; *H.verrucosa*: UME-29336a.

**Figure 2.**
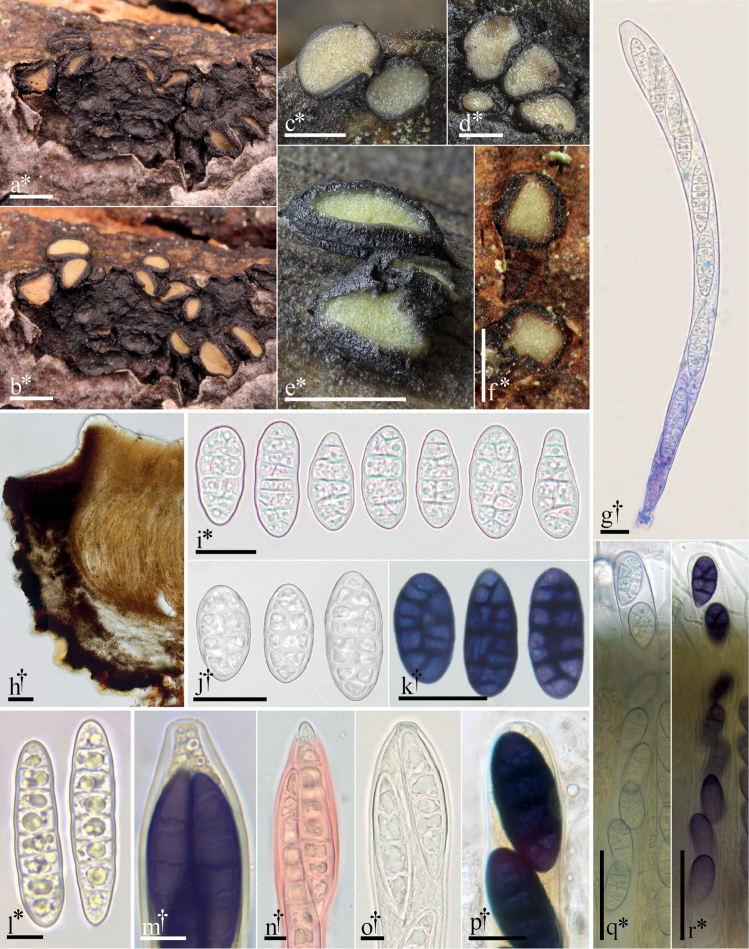
Morphological features of *Pseudographis*. **a–e, g, l–o***Pseudographispinicola***a** dried apothecia on bark **b** same apothecia hydrated **c–e** hydrated apothecia **g** dead ascus containing living ascospores (cb), **l** ascospores **m** ascus containing mature ascospores, detail of apex (in dilute L) **n** ascospore emerging from ascus apex (Cr) **o** ascus apex. **f, h–k, p–r***Pseudographiselatina***f** hydrated ascomata **h** 15 µm thick longitudinal section **i–j** ascospores **k** ascospores (in dilute L) **p** detail of ascus apex (L) **q** turgid ascus **r** same ascus (in dilute L). All microphotographs of cells and tissues mounted in water unless otherwise noted: cresyl blue (cb), Congo red (Cr), Lugol’s solution (L). † = dead, * = living. Scale bars: 1 mm (**a–f**); 50 µm (**h, q–r**); 20 µm (**i–k**); 10 µm (**g, n–p**); 5 µm (**l–m**). Specimens photographed: *P.pinicola*: **a–b, g, l–o**, FH-18061706; **c–e** courtesy of Adam Polhorský; *P.elatina*: GJO-0090016.

Immature apothecia are closed, superficial, pulvinate bodies that open prior to maturity (hemiangiocarpous). In early developmental stages the monolocular centrum consists of paraphysoids that are soon replaced by paraphyses immersed in a gel. The excipulum is stromatic and highly melanized. Asci are elongate-cylindrical, unitunicate, and do not react in iodine-based reagents. Ascus apices are undifferentiated or possess a ± reduced apical ring. The walls of discharged asci are often distinctly transverse-striate or wrinkly. Ascospores are large, elongated and transverse-septate or ellipsoid and muriform, hyaline, and lack a gelatinous sheath. In ascospores of *Pseudographis* species the cell wall reacts opaque dark blue to dark purple in iodine-based reagents (Figs 1, 2). Conidial states are unknown (Magnes 1997: 5, 27).

Eriksson (1992) erected Triblidiales to accommodate Triblidiaceae. Magnes (1997: 5–6) classified the family within Rhytismatales M. E. Barr ex Minter (Leotiomycetes), considering Triblidiales a synonym. Rhytismatales encompasses non-lichenized, saprobic or parasitic to pathogenic fungi. Members of the order typically produce desiccation-tolerant ascomata with heavily melanized stromatic covering layers that close in arid conditions. The “tar-spot” fungus, *Rhytismaacerinum*, a foliar parasite of *Acer* spp., is arguably the most frequently encountered member of the order in Europe and North America; it is the type species of the order.

Triblidialean fungi are associated with plant genera in three families in the Northern Hemisphere: Pinaceae, Ericaceae, and Fagaceae (Magnes 1997: 16). A few collections are known from the Southern Hemisphere on *Nothofagus* (Nothofagaceae) in Chile. They typically inhabit elevated substrates: bark and (more rarely) decorticated wood of living and dead trees. Such substrates represent harsh ecological niches that are exposed to high solar radiation, wind, and extreme fluctuations in humidity and temperature, as well as being nutrient poor (Sherwood 1981: 17, 19). Triblidialean fungi are considered to be saprobes, and Magnes (1997: 28) speculates an endophytic state exists in *Triblidiumcarestiae* (De Notaris) Rehm and *T.hafellneri* Magnes that grow on dwarf Ericaceae.

Triblidialean fungi are distributed within temperate and boreal forests. They are known primarily from the Northern Hemisphere, from lowland to subalpine elevations, though Magnes (1997: 16–17) cited a paucity of collection data related to actual distribution of these fungi. Many taxa are known from Central and Northern Europe, but the occurrence of these in similar climatic and ecologic regions in North America remains unclear due to limited collections from this continent. Though the centers of distribution of *Rhododendron* and Pinaceae occur in China, triblidialean fungi from this country are only represented by *Huangshaniaverrucosa* O. E. Eriksson (Magnes 1997: 16–17).

The occurrence of mature, sporulating apothecia of triblidialean fungi are not restricted to a particular season in the Northern Hemisphere. We surveyed collection dates of specimens of *Triblidium* and *Pseudographis* that were studied by Magnes (1997) and observed that collections were made in very nearly every month of the year for both genera. *Huangshania* is known only from type and authentic material of *H.verrucosa* collected in central China in November and from type material only of *H.novae-fundlandiae* (Rehm) Magnes collected in eastern Canada in February. Additionally, our own collections of various *Triblidium* and *Pseudographis* species were made in February to September.

Our primary aim in conducting this research is to evaluate Magnes’s (1997) emendation and classification of Triblidiaceae in Rhytismatales using a molecular phylogenetic approach. Our results facilitate a phylogenetically informed interpretation of the unique and distinctive ascomatal morphologies that have led, in part, to such confusion in the classification of these fungi at ordinal and familial ranks. The inclusion of triblidialean fungi in Rhytismatales brings to light instances of ascospore morphologies that are more commonly observed among lichenized and non-lichenized taxa in Lecanoromycetes and Dothideomycetes. We have generated 27 new gene sequences from nine species, some of which represent taxa that have not been previously sampled. This data will enrich future systematic and metagenomic studies.

We conclude our Introduction with a history of Triblidiaceae Rehm sensu Magnes. In Results, we present our three-gene phylogeny, as well as a taxonomic section with an emended taxonomy and a description of Triblidiaceae. We also provide a note including the salient features of *Pseudographis* that is intended to supplement the description of Rhytismataceae Chevallier given by Baral (in Jaklitsch et al. 2016: 192–194). We conclude with a discussion on occurence of triblidialean fungi, their trophic state, and ascospore morphology as related to their ecological significance.

### History of Triblidiaceae Rehm

**Notes**

The following history is adapted and expanded from Magnes (1997: 7–9) who provided a concise table of the various circumscriptions of Triblidiaceae since the origin of the family (p. 9). Throughout, we have adopted the exact spelling of taxa used in the literature under discussion. Regarding Rehm (1887–1896) in particular, it is worth noting that the spelling of the same taxon varies frequently (e.g. “Triblidieae” and “Tryblidieae”). Citations of Rehm (1887–1896) are complicated in that the publication was issued in parts over a number of years; we are using exact publication dates in our citations based on Stafleu and Cowan (1983: 476–478). The current classification of various taxa follows Jaklitsch et al. (2016). We conclude with a summary of Magnes’s contribution to the study of the family and some recent findings.

**Orthography and etymology**

Triblidiaceae is the correct spelling of this family. The name is based on the generic name *Triblidium* and a single species, *T.caliciforme* (Rebentisch 1805: 40). Rehm (1888: 196) provided the following etymologic and orthographic note: “Tryblidium stammt von τρύβλιον, die Schale, und ist demgemäss zu schreiben.” The Greek term “τρύβλιον” can be transliterated to *tryblion* (Latin: *tryblium*) (Brown 1956: 244; Stearn 2010: 253–254) and translates to English as *a cup, bowl* (Liddell and Scott 1875: 1664). Fries (1823: 183) used the same Greek word. The Greek suffix –*idium* indicates smallness (Stearn 2010: 296). Article 60.1 and specifically article 60.4 of the current nomenclatural code (Turland et al. 2018) allow for the use of “y” in scientific names though it is foreign to classical Latin. Regardless, Article F.3.2 states that the spelling used in a sanctioning work is treated as conserved. *Triblidium* is used consistently in the sanctioning works by Fries (Article F.3.1) (i.e. Fries 1823: 183, 1828: 130–131, 1832: 193). Rehm (1888: 191) erected Triblidiaceae as “Tryblidiaceae”, and it is clear from the etymological note and a correction to replace the suborder name “Triblidieae” with “Tryblideae” on page 99 (Rehm 1896: [1271]) that he intended a consistent orthography that included “y.” Regarding the spelling of the epithet, which has been used as *caliciforme* and *caliciiforme*, Rebentisch (1805: 40) stated in the protolog that this *Triblidium* species resembles *Caliciumsphaerocephalum*, though it is larger. The correct spelling of the epithet “*caliciiforme*” is thus obtained from the stem of the generic name *Calicium*, to which the connecting vowel *i* is added (Article 60.10 (a and b), ex. 35 in Turland et al. 2018) followed by the neutral suffix -*forme* to indicate similarity in form. Therefore, the correct spelling of this taxon is *Triblidiumcaliciiforme*.

**Rehm’s Triblidiaceae and classification**

Rehm’s higher-rank classification for class Ascomycetes included two orders based on the gross morphology of ascomata: Hysteriaceae and Discomycetes (Rehm 1887–1896). Order Hysteriaceae was characterized by black, membranous to carbonaceous, oblong ascomata that open at maturity by a longitudinal slit (Rehm 1887: 1). These represented a transition between members of Pyrenomycetes and Discomycetes. The genera included are currently placed in Dothideomycetes, such as *Hysterium*, *Glonium*, and *Acrospermum*, and more disparate fungi currently placed in Leotiomycetes, such as *Hypoderma* and *Lophodermium* (both Rhytismatales). Discomycetes encompassed club-, cup-, dish- and lens-shaped ascomata that support a hymenium that is entirely exposed at maturity (Rehm 1887: 56). Discomycetes was divided into two principal groups: Pezizaceae, with cup-shaped apothecia that initially develop closed but open widely at maturity to reveal a flat hymenium, and Helvellaceae that produces larger, fleshy, stipitate ascomata with a hymenium that is exposed during development (Rehm 1887: 59). Pezizaceae consisted of five suborders: Phacidiaceae, Stictideae, Triblidieae [Tryblidieae], Dermateaceae, and Pezizeae (Rehm 1887: 59–60). According to Rehm, the apothecia of members of suborder Tryblidieae develop within the substrate, becoming erumpent and finally sessile. These are pulvinate and substipitate, with a membranous or horny exterior. At maturity lobes of covering tissue pull away to reveal the disc. Suborder Tryblidieae included Rehm’s new families Tryblidiaceae and Heterosphaerieae (containing *Heterosphaeria*, *Odontotrema* and *Scleroderris* [= *Godronia*]), with Tryblidiaceae distinguished by solitary, round to lentiform, black apothecia that open with thick, torn margins. The family included two genera: *Tryblidium* Rebentisch and *Tryblidiopsis* P. Karsten (Rehm 1888: 191).

Regarding *Pseudographis*, Rehm (1888) placed the genus in Discomycetes, suborder Phacidiaceae. This taxon was subdivided into families Pseudophacidieae and Euphacidieae. Species comprising both families produced ascomata that were immersed within the substrate in early development. They differed in that ascomata of Pseudophacidieae species emerged from the surrounding host tissues by splitting them aside and did not remain covered by a thin layer of host cells at maturity. The gross texture of their excipular tissues were membraneous or carbonaceous while those of Euphacidieae were only membranous (e.g. *Phacidium*, *Coccomyces* and *Rhytisma*, among others) (Rehm 1887: 60). Pseudophacidieae included *Pseudographis*, along with *Pseudophacidium*, *Clithris* [= *Colpoma*], *Cryptomyces* (all Leotiomycetes) and *Dothoria* (Dothideomycetes), among others. Rehm treated both *P.elatina* and *P.pinicola*, noting the affinities of these to members of his suborder Tryblidieae, but he retained them in Pseudophacidieae due to differences in development that he perceived (Rehm 1888: 99). However, in his Additions (1896: 1249) Rehm relegated the type species of the genus, *P.elatina*, to synonymy under *Tryblidiummelaxanthum*. The complete history of *Pseudographis* is given in Magnes (1997: 62–63).

In a later work Rehm (1904: 522–526) summarized his dealings with certain nomenclatural acts that had confused the circumscription of Triblidiaceae. De Notaris (1847: 15–16) had created a later homonym of *Triblidium* based on *T.hysterinum*Dufour (1828: 321) [= *Rhytidhysteronhysterinum*]. He then created a younger synonym, *Blitridium* (anagram of *Triblidium*), to accommodate *Triblidiumcaliciiforme* Rebentisch (De Notaris 1864: 374). Saccardo, against the principle of priority, adopted De Notaris’s *Triblidium* (Saccardo 1883: 740) and *Blitridium* (Saccardo 1889: 802). Rehm (1888: 196–198), observing the rule of priority, relegated *Blitridium* to synonymy under *Triblidium* Rebentisch. Likewise, he synonymized de Notaris's *Triblidium* under *Tryblidiella*Saccardo (1883) [= *Rhytidhysteron* Spegazzini (1881)] (Rehm 1889: 233).

In his last summarizing work, Rehm (1912: 137–139) treated Triblidiaceae with a revised circumscription to include not just *Tryblidiopsis* and *Tryblidium*, but also *Pseudographis* and *Tryblidiella*. This circumscription followed Höhnel (1909: 73, 1910: 2) who discussed *Tryblidium*, *Tryblidiopsis* and *Pseudographis* and who recognized Tryblidiaceae to contain the former two genera. In Höhnel’s opinion, Tryblidiaceae and Pseudophacidiaceae (that contained *Pseudographis*) were sister taxa, as evidenced by their similarities in developing within host tissues and then finally becoming erumpent and sessile at maturity. He perceived that both families were entirely similar in ascomatal structure but that they were not typical Discomycetes due to differences in centrum development and ascomatal dehiscence, thus they formed a transitional link to Dothideaceae (Höhnel 1909: 73). Later, Höhnel (1910: 2) explicitly placed *Pseudographis* within Tryblidiaceae.

**Höhnel’s widening circumscription of Triblidiaceae**

From his observations of the structure of the sterile tissues of apothecia, Höhnel (1918: 145–146, 154) expanded his circumscription of Triblidiaceae beyond *Tryblidium*, *Tryblidiopsis* and *Pseudographis* to include other, unrelated genera: *Crumenula* [= *Crumenulopsis*] (Leotiomycetes, Helotiales), *Melachroia* [= *Podophacidium*] (Leotiomycetes, Helotiales*incertae sedis*) and *Tryblidiella* [containing *Rhytidhysteron* (as *Rhytidhysterium*)]. In a posthumously published paper, Höhnel (1924: 68, 70) elaborated on the structure of the apothecial tissues that he considered to hold taxonomic significance, namely the hypothecium (medullary excipulum) that he described as thick and composed of “thick-walled, cartilaginous, densely plectenchymatic intertwined hyphae.” He then expanded Triblidiaceae to encompass yet more unrelated genera including: *Asterocalyx* (Leotiomycetes, Helotiales*incertae sedis*), *Tympanis* (Leotiomycetes, Phacidiales, Tympanidaceae), *Godronia* (Leotiomycetes, Helotiales, Godroniaceae), *Scleroderris* [= *Godronia*] and *Cenangium* (Leotiomycetes, Helotiales, Cenangiaceae).

**Nannfeldt’s treatment**

Nannfeldt (1932: 312, 331) did not conduct an in-depth study of *Tryblidium* due to a lack of sufficient material and treated the genus only briefly. He made no recommendations regarding its classification apart from noting that the apothecial structure indicated a close relationship to Helotiales and their similarities with *Tryblidiopsis* and *Heterosphaeria*. Nannfeldt commented that the muriform ascospores of *Tryblidium* were unique in Helotiales. He considered *Pseudographis* to be a link between Discomycetes and apothecial lichens and placed this genus in Lecanorales due to the epithecium and asci that he interpreted as thick-walled.

**Sherwood and Hawksworth’s classification**

Hawksworth and Sherwood (1982: 264) commented on the relationship between Odontotremataceae and Triblidiaceae. Odontotremataceae are minute saprobic or lichenicolous inoperculate discomycetes, often with melanized excipular tissues and dentate margins, and with iodine-negative asci with thickened apices that are pierced by a pore. The authors noted that the asci of Triblidiaceae and Graphidaceae (Ostropales) also share this feature. Furthermore, the “reddish-purple” reaction of *Pseudographis* ascospores in iodine reagents suggested affinities between Triblidiaceae and Graphidales [= Ostropales]. They circumscribed Triblidiaceae to include *Pseudographis* and *Triblidium*, but removed *Tryblidiopsis* to Rhytismatales. Thus, Triblidiaceae was placed within Ostropales (Hawksworth et al. 1983: 273–274; Sherwood-Pike 1987: 139, 141).

Hawksworth and Sherwood’s (1982: 264) aforementioned observation regarding ascus morphology in Triblidiaceae was discussed by Eriksson (1992: 6–7) who commented that younger asci generally appear to have thicker walls than those that are mature because they are not fully extended and turgid. This is a developmental rather than a taxonomic character, in contrast to the mature asci of true ostropalean fungi wherein the apices are actually thickened. Furthermore, Hawksworth and Sherwood were almost certainly basing their statements on observations made from preserved material, wherein all of the asci are dead and would show thickened walls, a phenomenon comprehensively reviewed by Baral (1992: 351–352, figs 6–10).

Magnes (1997: 8–9) observed that Hawksworth and Sherwood (1982) did not comment on the type of interascal filaments (paraphysoids/paraphyses) in Triblidiaceae. He concluded that as the authors considered the family to be closely related to Odontotremataceae (that have paraphyses only), they must have regarded the interascal filaments of Triblidiaceae as paraphyses.

**Eriksson describes *Huangshania* and classification of Triblidiaceae in Triblidiales**

Eriksson (1992) addressed the relationships of Triblidiaceae in the course of describing *Huangshania*. His circumscription of the family followed that of Hawksworth and Sherwood (1982) with the addition of *Huangshania* to *Triblidium* and *Pseudographis*. He noted that similarities in ascomatal structure across the three genera indicated a natural group. Eriksson and Hawksworth (1991: 44) maintained Triblidiaceae within Graphidales with uncertainty. However, with his newly circumscribed family, Eriksson revaluated this classification. He considered a number of orders belonging to three classes that possessed apothecioid ascomata and angiocarpous/hemiangiocarpous modes of development in which to place Triblidiaceae. One group of orders consisted of primarily lichenized taxa in Lecanoromycetes: Graphidales [= Ostropales], Gyalectales [= Ostropales] and Peltigerales. Another group consisted of primarily non-lichenized taxa in Leotiomycetes: Lahmiales, Leotiales, and Rhytismatales. The final order, Patellariales, consist of bitunicate, non-lichenized, apothecioid taxa in Dothideomycetes (Eriksson 1992: 8–9; Jaklitsch et al. 2016). Eriksson considered that *Graphis*, the type genus of Graphidales, shared a morphological character with *Pseudographis* in ascospores that turn blue in iodine reagents. However, *Graphis* species differed from those of *Triblidium* in thicker walled asci with thickened apices and well-differentiated pore structures. Eriksson considered Triblidiaceae to have affinities with members of Graphidales, but the family differed in ascus wall structure and centrum morphology. Therefore, it was prudent to erect a separate order: Triblidiales. This would have the added benefit of maintaining a more homogenous circumscription of Graphidales (Eriksson 1992: 8–9).

**Triblidiaceae Rehm sensu Magnes**

Based on Rehm’s (1912) circumscription, Magnes (1997) comprehensively revised Triblidiaceae. We found only four names involving *Triblidium* and *Pseudographis* that he did not treat. He described two new species of *Triblidium* and a variety of *P.elatina*. Species excluded from Triblidiaceae were assigned to 22 genera belonging to seven orders (Magnes 1997: 5–6).

Magnes’s circumscription of Triblidiaceae followed Eriksson’s (1992). Eriksson had rejected placing Triblidiaceae in Rhytismatales because he considered paraphysoids not to be present in this order, though he noted similarities in ascus structure (Eriksson 1992: 9). Magnes claimed that paraphysoids were in fact present in certain genera of Rhytismataceae. He cited literature and noted his own observations of these structures in *Tryblidiopsispinastri*. Therefore, based on similarities in ascomatal development and ascus structures, he hypothesized that Triblidiaceae formed an isolated group within Rhytismatales and classified the family within this order, relegating Triblidiales to synonymy. Furthermore, Magnes (1997: 17) suggested a close relationship between Triblidiaceae and Rhytismataceae, particularly with members of *Coccomyces* (citing Sherwood 1980). He rejected a close relationship between Triblidiaceae and Graphidaceae due to differences in spore wall development (citing Sherwood 1977: 12, fig. 2) and different ascus structures.

**A molecular phylogeny of Rhytismatales**

Important research in the systematics of Rhytismatales was conducted by Lantz et al. (2011). They elucidated a core clade of Rhytismatales sensu stricto, and demonstrated that ascoma and spore shape, characters used in traditional morphology-based delimitation of genera, were generally unreliable. However, the authors noted that these could be useful in some cases, when combined with other characters (p. 57). Notably, two strongly supported clades were observed in their two-gene molecular phylogeny that included 91 species. The clades were informally termed “radiate” and “bilateral.” The radiate clade encompassed species that produce circular ascomata opening in a radiate fashion to form tooth-like processes to expose the disk, versus species that produce hysterioid or otherwise elongated ascomata that open in a bilateral fashion along a single, longitudinal slit.

## Materials and methods

### Specimens

**Collection methods and handling of fresh specimens**

Triblidialean fungi are best collected in humid weather as the disc is exposed, making apothecia more visible. Alternatively, dry ascomata can be sprayed with tap water. Specimens of triblidialean fungi were placed in paper bags, allowed to air-dry, and stored in a cool, dry location in the laboratory. Specimens may remain alive and capable of sporulation when rehydrated up to a month or possibly more after collection. Although asci may not discharge after this time, ascospores may remain viable within asci for a lengthy period.

**Fungarium specimens**

Because specimens of triblidialean fungi are often small, only two apothecia were removed from any given specimen: one for morphological analysis and the other for DNA extraction. In particular cases, we used only one apothecium for both of these purposes.

### Morphological data collection

**Macrophotography of ascomata**

Samples of substratum bearing apothecia were hydrated with a spray bottle containing tap water. Macrophotographs were made in the laboratory with a Canon EOS 60d digital SLR camera mounted to a height-adjustable camera support mounted on a table. Macrolenses included either a Canon EF-S 60 mm or a Canon MP-E 65 mm with an attachable ring light. Subjects were photographed against dark-gray or black matboard.

**Microphotography and analysis of digital microphotographs**

We employed a laboratory-dedicated Olympus SZX9 stereomicroscope or an Olympus BX40 compound light microscope with an Olympus XC50 5.0 megapixel digital camera and Olympus cellSens Standard 1.14 image processing software, calibrated to these optical devices. Austrian specimens collected and studied by G.F. were examined using an Olympus SZX10 stereomicroscope and an Olympus BX51 compound light microscope. Images and data were gathered with an Olympus DP72 digital camera and measurements were made with an eyepiece reticle or with Olympus cellSens Dimension software.

**Mounting media, stains and reagents for compound light microscopy**

In all cases, tap water was used as a mounting medium. Material for crush-mounts or sectioning was wetted in dilute ammonia or in 70% ethanol and then rehydrated in tap water. Ammoniacal or SDS Congo red were used to stain cell walls. Cresyl blue, phloxine, cotton blue in lactophenol, and Lugol’s and Melzer’s iodine reagents were used to stain cell contents. Living cells were stained with cresyl blue or dilute Lugol’s. Three-percent potassium hydroxide (KOH) was used as a mountant in crush mount or section preparations in order to facilitate separation of cells. This reagent was also used to pretreat asci for subsequent treatment with Lugol’s and Melzer’s. Analysis of living and dead cells, as well as the use of various mounting media and iodine-based reagents in fungal taxonomy, follows Baral (1987, 1992), Largent et al. (1977) and Leonard (2006).

**Observations of living, mature ascospores**

These were studied from ascospore deposits and from hand-sections or squash mounts. Ascospores obtained from deposits may show a large variation in size. For this reason, we also measured mature-looking ascospores still inside asci and noted the number of ascospores per ascus. Ascospore deposits were obtained by placing a cover-glass over sufficiently hydrated apothecia in a Petri dish lined with moist filter paper and sealed with Parafilm. The progress of ascospore accumulation was monitored under high magnification with a stereomicroscope by focusing down onto the undersurface of the cover-glass. Ejected ascospores appear as small, gem-like, shining bodies suspended in small droplets of condensation. After a period of one hour to overnight, the cover-glasses were carefully removed with forceps and gently placed on a small droplet of tap water or other reagent on a microscope slide.

**Structure and tissues of apothecia**

In addition to crush-mounts of various apothecial tissues, we prepared longitudinal sections of apothecia by hand-sectioning or by using a freezing stage microtome. Hand-sections were prepared from hydrated specimens under magnification with a stereomicroscope. One-half of a double-sided razor was repeatedly drawn across the median area of an apothecium. Use of a freezing stage microtome allowed uniformity in thickness. Material sectioned in this way is dead. Pieces of substratum supporting an apothecium were hydrated, soaked in a solution of dilute gum arabic, and oriented on an electric, water-cooled freezing stage (Physitemp BFS-5MP) mounted to a sliding microtome. Additional dilute gum arabic matrix was then added to completely envelop and support the tissue during sectioning. Sections were cut to approximately 15–25 µm and were removed from the blade with a fine-point paintbrush to water on a microscope slide. Sections of apothecia that were more or less the greatest width were representative of the middle of the apothecium. These were preferred for light microscopy. The remaining sections were air-dried and placed in a microscope slide packet to be kept with the specimen. This technique is outlined in Dring (1971: 103–105), Gray (1954: 157–160) and Sass (1958: 93–94). Sections made in this way greatly facilitate histologic analysis and yield publication-quality microphotographs. Furthermore, this technique follows Sherwood (1977, 1980) for ostropalean and rhytismatalean fungi. Analysis and descriptions of apothecial structure and fungal tissue type designations follow Korf (1973).

### Cultures

**Culture media**

Difco potato dextrose agar (PDA) and Difco malt extract agar (MEA) were used to cultivate mycelium for DNA extraction. These media were prepared according to the manufacturer’s instructions with deionized water and autoclaved for 15 m (121 °C, 19 psi), cooled, and poured into 60 × 15 mm polystyrene Petri dishes in a laminar flow hood.

**Inoculation of media**

Polysporous cultures were established by means of ascospore deposition directly onto PDA or MEA. A piece of substratum bearing 1–3 air-dried apothecia was excised from the sample under a stereomicroscope. It is important to not rehydrate air-dried specimens prior to this step as discharged ascospores may be lost. This is especially important in triblidialean fungi with large ascospores, and where a small number of mature asci are present. Care was taken to cut only around these apothecia and to not include any other sporomata that may be incidentally present. The apothecia were then placed on a small piece of well-dampened filter paper. A Petri dish with PDA or MEA was inverted so that the surface of the media faced down. The bottom of the dish was tilted up slightly and, using forceps, the hydrated paper and apothecia were carefully inserted and placed on the inner surface of the lid. The dish was wrapped in Parafilm. A circle was drawn in alcohol-soluble marker on the bottom of the dish over the apothecia. This served to demarcate where the ascospores should land on the media. The progress of the ascospore print was checked every hour under a stereomicroscope as described above. When sufficient ascospores had accumulated, the paper and apothecia were carefully removed and the plate resealed with Parafilm. The apothecia were dried and placed in a small, appropriately labeled packet so that they might be reexamined if necessary. The surface of the inoculated media was checked daily for 7 days to monitor ascospore germination and growth of any fungal or bacterial contaminants. As PDA and MEA are transparent, this was accomplished by placing the inverted Petri dish on a compound light microscope stage and scanning with the 4× and 10× objectives. Cultures were stored in an incubator at 22 °C in darkness.

### Phylogenetic analysis

**Sampling**

We sampled approximately 100 mg of living mycelium from pure cultures. These samples were stored at −80 °C until DNA extraction was performed. Since apothecia of triblidialean fungi in fungarium specimens are typically sparse, only one or one-half of an apothecium was removed from any given specimen for DNA extraction.

**DNA extraction**

Samples from pure cultures were processed using Qiagen (Germantown, Maryland) DNeasy Plant Mini Kit according to manufacturer protocols. Fungarium specimens were processed using Qiagen QIAmp DNA Micro Kit according to manufacturer protocols with a 12–24 hour cell-lysis period in an agitating hybridization oven set to 56 °C.

**DNA amplification and sequencing**

Undiluted DNA extracts, as well as 1/10 and 1/100 dilutions were used as templates. Polymerase chain reaction (PCR) amplification of ribosomal DNA (rDNA) included the nuclear internal transcribed spacer region (ITS) that is composed of the non-coding regions ITS1 and ITS2 that flank the gene encoding the 5.8S subunit, the nuclear large subunit (LSU), and the mitochondrial small subunit (mtSSU). The choice of gene regions for PCR followed Lantz et al. (2011) with the inclusion of the ITS region (Schoch et al. 2012). Primers for ITS included ITS1F (Gardes and Bruns 1993) and ITS4 (White et al. 1990). For recalcitrant isolates or those extracted from old specimens, internal primers ITS2 and ITS3 (White et al. 1990) were employed to target shorter ITS fragments for amplification. Primers for mtSSU were mrSSU1, mrSSU2, mrSSU2R and mrSSU3R (Zoller et al. 1999). Primers for LSU were LR0R: 5’– ACCCGCTGAACTTAAGC–3’ and LR3R: 5’– GTCTTGAAACACGGACC–3’ (Vilgalys 2018), LR3 and LR6 (Vilgalys and Hester 1990). For each PCR reaction (25 µL) we used Lucigen (Middleton, Wisconsin) EconoTaq polymerase and EconoTaq 10x buffer. Each reaction mix was composed as follows: 0.125 µL polymerase, 1.25 µL 10 mM of each primer pair, 0.5 µL 10 mM dNTP, 2.5 µL buffer, 1.25 µL dimethyl sulfoxide (DMSO), 1.25 µL 1% bovine serum albumin (BSA), 11.875 µL dH20 and 5 µL DNA template in solution. The addition of BSA and DMSO followed Farell and Alexandre (2012). The optimum annealing temperature (T_a_) in °C for each primer pair was determined using IDT (Coralville, Iowa) OligoAnalyzer (IDT 2019). The sequence of one primer is analyzed at a time and the following concentration parameters for the reaction mix listed above are also entered: oligonucleotide: 0.5 µM; Na+: 50 mM; Mg++: 1.5 mM; dNTP: 0.2 mM. The T_a_ was obtained by subtracting 5 °C from the lowest melting temperature of the two primers. Thermocycler PCR profiles are as follows: for ITS, initial denaturing at 95 °C for 2 min followed by 35 cycles of denaturing at 95 °C for 1 min, annealing at (T_a_) for 1 min and extension at 72 °C for 45 sec, with a final extension step of 72 °C for 10 min; for LSU, initial denaturing at 94 °C for 2 min followed by 40 cycles of denaturing at 94 °C for 1 min, annealing at (T_a_) for 1 min and extension at 72 °C for 1:30 min with a final extension step of 72 °C for 7 min; for mtSSU, initial denaturing at 94 °C for 3 min followed by 40 cycles of denaturing at 94 °C for 1 min, annealing at (T_a_) for 1 min and extension at 72 °C for 1 min with a final extension step of 72 °C for 7 min. PCR products were visualized via gel electrophoresis using 1% gel agarose, stained with Biotium (Fremont, California) GelRed nucleic acid stain and visualized with UV light. PCR product cleanup, sequencing reactions and Sanger sequencing were performed by GENEWIZ sequencing service (Cambridge, Massachusetts) using the same primer pairs as in the PCR reactions. When necessary, PCR products were purified with New England BioLabs (Ipswich, Massachusetts) Monarch PCR & DNA Cleanup Kit (5 µg) or Qiagen QIAquick Gel Extraction Kit using Biotium GelGreen nucleic acid stain.

**Handling sequence data**

Sequences were edited in Geneious (v. 6.1.7) (Kearse et al. 2012) or Sequencher 5.1 (Sequencher 2012). A BLASTn (Altschul et al. 1990) search was used to verify the sequence as originating from the intended organism and to identify closely related sequences for inclusion into the dataset as a subject. Sequences were accessioned into GenBank (Clark et al. 2015) citing the respective collection numbers from which they originated. These GenBank accession numbers are listed in Table 1 along with additional sequences downloaded from GenBank that were combined into the dataset. A footnote providing information on the origin and other pertinent information is provided for each of these sequences.

**Table 1.** Specimens used in this study with family, order, voucher/strain number and GenBank accession numbers. New sequences of *Triblidium*, *Huangshania* and *Pseudographis* are indicated in bold.

**Order**	**Family**	**Species**	**Voucher or strain**	**ITS**	**LSU**	**mtSSU**
Geoglossales	Geoglossaceae	*Trichoglossumhirsutum*	AFTOL-ID 64	DQ491494	AY544653	AY544758
Cyttariales	Cyttariaceae	*Cyttariadarwinii*	Isolate 57	NA	EU107206	EU107235
		*Cyattariahariotii*	Isolate 55	NA	EU107218	EU107246
		*Cyttariaexigua*	Isolate 77	NA	EU107214	EU107240
Erysiphales	Erysiphaceae	*Blumeriagraminis*	?	AB000935	AB022362	NA
		*Arthrocladiellamougeotii*	?	AF073358	AB022379	NA
Helotiales	Helotiaceae	*Cudoniellaclavus*	AFTOL-ID 166	DQ491502	DQ470944	FJ713604
	Lachnaceae	*Erioscyphellaabnormis*	KUS F52080 (7)	JN033395	JN086698	JN086772
Leotiales	Leotiaceae	*Leotialubrica*	AFTOL-ID 1253	DQ491484	AY544644	AY544746
		*Microglossumolivaceum*	FH-DSH97-103	AY789398	AY789397	NA
		*Microglossumviride*	SAV 10249	KC595263	KC595264	NA
Medeolariales	Medeolariaceae	*Medeolariafarlowii*	DHP # 07-637	GQ406809	GQ406807	NA
Phacidiales	Phacidiaceae	*Phacidiumlacerum*	AFTOL-ID 1253	KJ663841	DQ470976	FJ190623
		*Pseudophacidiumledi*	Lantz 366 (UPS)	NA	HM140563	HM14383
		*Potebniamycespyri*	S001	DQ491510	DQ470949	NA
Rhytismatales	Cudoniaceae	*Spathulariaflavida* 1	KUS-F52331	JN033405	JN086708	JN086781
		*Spathulariaflavida* 2	CBS 399.52	NA	AY541496	AY575101
		*Cudoniaconfusa*	M. Carbone 312	KC833165	KC833216	NA
		*Cudoniacircinans*	Lantz & Widén 402 (UPS)	EU784190	HM140551	HM143791
	Rhytismataceae	*Coccomycesdentatus*	OSC 100021	DQ491499	AY544657	AY544736
		*Coccomycesleptideus*	Lantz 393 (UPS)	NA	HM140506	HM143783
		*Hypodermacordylines*	?	JF683420	HM140521	HM143795
		*Hypodermarubi*	ICMP 17339	JF683419	HM140526	HM143801
		*Hypohelionscirpinum*	Lantz 394 (UPS)	NA	HM140531	HM143806
		*Lophodermiumeucalypti*	ICMP 16796	EF191235	HM140541	HM143817
		*Rhytismaacerinum*	?	GQ253100	FJ495190	HM143837
	Marthamycetaceae	*Marthamycesquadrifidus*	ICMP: 18329	NA	HM140559	HM143832
		*Propolisfarinosa*	ICMP 17354 (8)	MH682229	HM140562	MH698451
		*Cyclaneusmaminus*	CBS 496.73	NR153910	FJ176868	FJ190629
		*Mellitiosporiumversicolor*	Lantz 357 (UPS)	NA	HM140560	NA
		*Naemacyclusculmigenus*	TNS: F-41728	AB745435	AB745437	AB745436
Thelebolales	Thelebolaceae	*Thelebolusglobosus*	AFTOL-ID 5016	NA	FJ176905	FJ190662
		*Thelebolusellipsoideus*	AFTOL-ID 5005	NA	FJ176895	FJ190657
Chaetomellales	Chaetomellaceae	*Chaetomellaoblonga*	CBS 110.76	AY487082	AY487083	NA
		*Pilidiumacerinum*	CBS 736.68	NR119500	AY487092	NA
		*Xeropilidiumdennisii*	TU104501	LT158441	KX090824	NA
Rhytismatales	Triblidiaceae	***Huangshaniaverrucosa***	UME-29336a	MK751793	MK751802	MK751716
		***Triblidiumcaliciiforme***	FH-15071105	MK751797	MK751806	MK751720
		***Triblidiumcaliciiforme***	CUP-18080101	MK751798	MK751807	MK751721
		***Triblidiumcaliciiforme***	E-00012551	MK751799	MK751808	MK751722
		***Triblidiumcaliciiforme***	E-00905002	MK751800	MK751809	MK751723
		***Triblidiumcaliciiforme***	GJO-0088904	MK751801	MK751810	MK751724
	Rhytismataceae	***Pseudographiselatina***	GJO-0090016	MK751794	MK751803	MK751717
		*Pseudographiselatina*	NCBI:txid1695903	Genome	Genome	Genome
		***Pseudographispinicola***	FH-18061706	MK751795	MK751804	MK751718
		***Pseudographispinicola***	FH-NB842	MK751796	MK751805	MK751719

**Phylogenetic analysis**

We performed the phylogenetic analysis using three different DNA regions (ITS, LSU, mtSSU) from representative species of the Leotiomycete orders Cyttariales, Erysiphales, Helotiales, Leotiales, Marthamycetales, Medeolariales, Phacidiales, Rhytismatales, Thelebolales and Chaetomellales, with Geoglossales as an outgroup. We generated 27 new sequences including three genera: *Triblidium*, *Pseudographis*, and *Huangshania*. For information about the taxa sampling see Table 1. The sequences were aligned using the L-INS-i algorithm for the ITS region, and G-INS-i algorithm for mtSSU and LSU (Katoh and Toh 2008) with MAFFT v. 7.017 (Katoh et al. 2002). The Gblocks program v. 0.91b (Castresana 2000) was used to identify and eliminate ambiguously aligned regions, using the following relaxed settings (Talavera and Castresana 2007): minimum number of sequences for a conserved or flanking position = 24; maximum number of contiguous non-conserved position = 10; minimum length of a block = 5; and gaps in an alignment column allowed in up to half the number of included sequences. The analyses were performed using the optimal model of nucleotide substitution identified with JModeltest (Posada 2008), based on the Akaike information criterion (Akaike 1974). Maximum likelihood (ML) and Bayesian Inference (BI) analyses were performed using Geneious v. 6.1.7. (Kearse et al. 2012). Bayesian inference analyses followed Quijada et al. (2014), only varying in the number of starting trees (10 million generations) and the tree sampling (every 1000^th^ generation) for BI analysis. Branch support in ML was inferred from 1000 rounds of bootstrap (BS) replicates. We only considered supported clades for ML those with bootstraps values ≥75% and with posterior probability (PP) values ≥0.95 (strongly supported) for BI. Phylogenetics trees were drawn with Geneious and artwork was prepared in Adobe Illustrator CS5.

## Results

**Figure 3.**
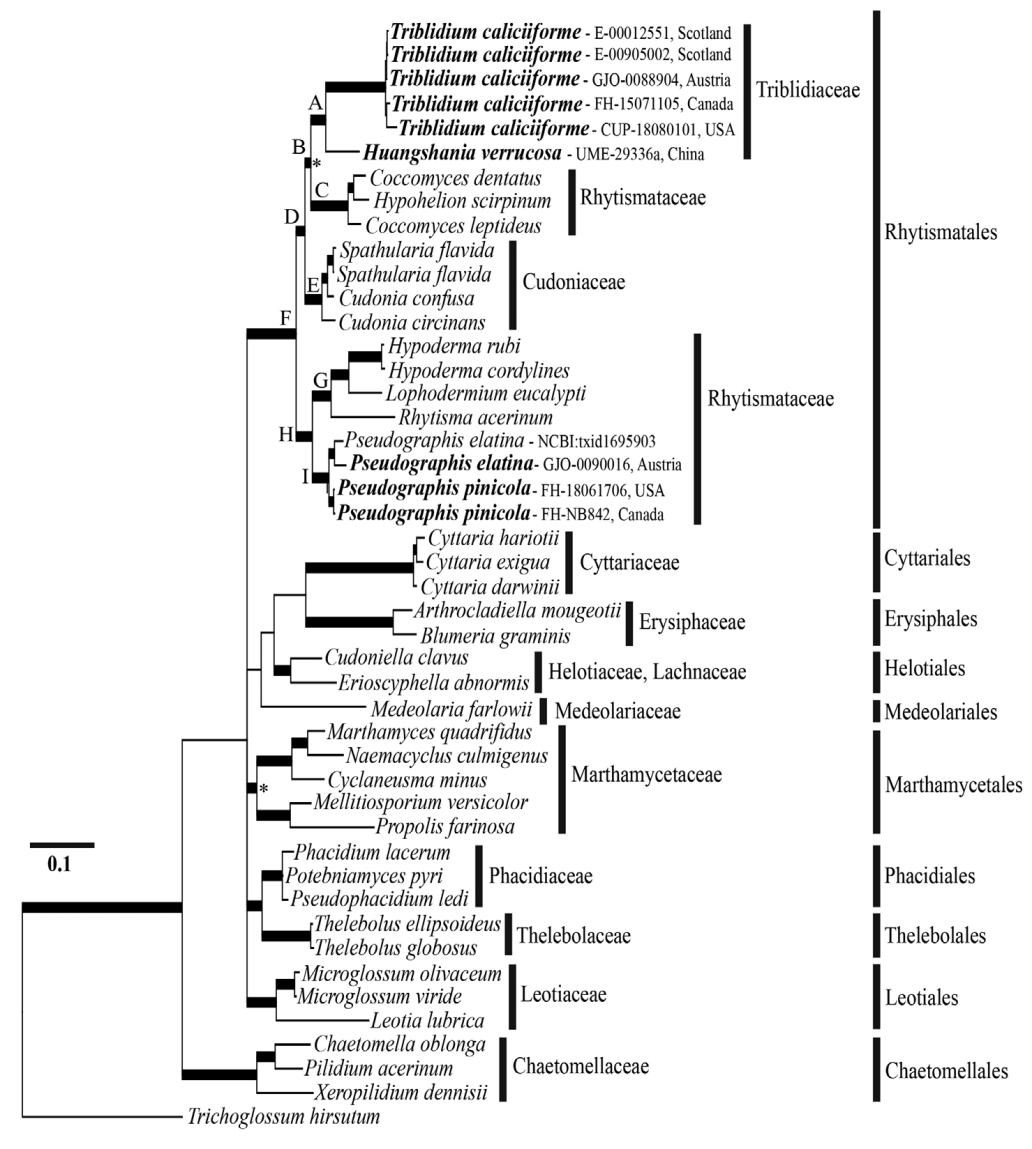
Bayesian majority-rule consensus tree of Leotiomycetes based on the ITS1-5.8S-ITS2 + LSU + mtSSU region. Thickened branches are those that were well supported by ML and BI methods. An asterisk indicates that this branch was supported only by Bayesian inference. Classification, orders and families follows Baral in Jaklitsch et al. (2016) using *Trichoglossumhirsutum* as the outgroup. Species for which molecular sequences have been generated for this study are given in bold followed by fungarium acronym, a dash, then identifying number.

Results from phylogenetic analyses of combined ITS, LSU and mtSSU DNA sequences are presented in Figure 3. Relationships among Leotiomycetes were investigated using three gene regions of 46 taxa that include 39 species in 30 genera, 13 families and 10 orders. The final alignment used for phylogenetic analysis contained 2229 bp (60% of the first alignment length), with 1280 variable and 908 parsimony-informative positions. The analyses identified 13 clades corresponding to 13 families and 10 orders of Leotiomycetes (Fig. 3). Rhytismatales (Fig. 3, clade F) contains eight important clades denoted by letters A–E and G–I. Rhytismataceae is polyphyletic (Fig. 3, clades C and H). Some species of *Coccomyces* and *Hypohelion* form a supported clade (Fig. 3, clade C: 100% MLBS, 0.99 BIPP) that corresponds to the radiate clade in Lantz et al. (2011). Within the radiate clade is a supported clade for Triblidiaceae, with *Huangshania* forming a lineage with *Triblidium* (Fig. 3, clade A: 100% MLBS, 1.00 BIPP). On the other hand, the two species of *Pseudographis* (*P.elatina* and *P.pinicola*) are in one supported clade (Fig. 3, clade H: 100% MLBS, 1.00 BIPP) distantly related to *Triblidium*. *Pseudographis* groups with other species in the bilateral clade (Lantz et al. 2011). This supported clade is represented here by some species of three different genera (*Hyphoderma*, *Lophodermium*, *Rhytisma*) (Fig. 3, clade G: 100% BIPP, 90.7 MLBS), notably containing the type species of Rhytismataceae, *R.acerinum*. The clade representing Cudoniaceae (Fig. 3, clade E) is sister to the radiate clade (Fig. 3, clade B) containing Triblidiaceae and certain members of Rhytismataceae.

### Taxonomy

#### Triblidiaceae

Taxon classificationFungiRhytismatalesTriblidiaceae

Rehm, Rabenh. Krypt.-Fl. ed. 2 (Leipzig), Band 1, Abth. 3: 191 (1888) emend. Karakehian

Figs 1 , 3, clade A

##### Type genus.

*Triblidium* Rebentisch: Fries, Index pl. berol.: 40 (1805); Syst. mycol. [Index]: 193 (1832).

##### Included genera.

*Triblidium* and *Huangshania*.

##### Position in classification.

Triblidiaceae, Rhytismatales, Leotiomycetes, Pezizomycotina, Ascomycota.

##### Description of Triblidiaceae.

**Ascomata** apothecia, scattered or in small clusters, primarily on bark of living or dead trees but occasionally also on decorticated wood, substratum not visibly degraded, without dark zone lines (Fig. 1a, b, e–g); primordia developing within the substratum, gradually emerging becoming superficial; young apothecia closed, pulvinate; excipulum stromatic and highly melanized (Fig. 1c, m), surface appearing dark brown-black, highly sculptured with coarse, polygonal areolae or ± cracked (Fig. 1a, b, f, g); in early developmental stages the monolocular centrum consists of paraphysoids (cf. Eriksson 1992: 5, fig. 5) that are replaced by paraphyses; apothecia rupturing the covering layer before full maturity (hemiangiocarpous) by several radial cracks, opening in humid conditions and closing when dry (Fig. 1a, b, f, g), persistent; approximately 0.6–1 mm high and 1–3 mm diameter; disk generally pale gray or brown or pale orange in some species (Fig. 1b, g). **Asci** elongate-cylindrical; apices ± hemispherical, undifferentiated, thin-walled, iodine negative (Fig. 1k, l, n, o), dehiscence via apical rupture; dehisced asci often with fine, transverse striations (Fig. 1u); spore number variable, generally 4–8 (Fig. 1c, d, n). **Paraphyses** narrow, filiform, apices flexuous, sparingly branched, hyaline, embedded in gel, lacking pigmented epithecium/exudate. **Ascospores** large, hyaline, ellipsoid to oblong-ellipsoid, muriform, smooth (*Triblidium*) or elongate-fusiform, transversely septate, smooth (in *H.novae-fundlandiae*) or coarsely verrucose (in *H.verrucosa*), iodine negative, appearing thick-walled when dead, lacking a gelatinous sheath (Fig. 1h–j, p–t). **Anamorph** unknown. **Trophic status**: presumed saprobes on woody plant hosts in Fagaceae, Ericaceae and Pinaceae. **Distribution**: mostly known from Northern Hemisphere, boreal and temperate forests (emended from Magnes 1997).

### Description of Rhytismataceae including characters of *Pseudographis* species

An expanded morphological description of this family includes characters of muriform ascospores (in *P.elatina*, Fig. 2i–k, p–r) and ascospore surfaces that react intensely dark blue/purple in iodine-based reagents (Fig. 2k, m, p, r) (Magnes 1997: 54; Baral in Jaklitsch et al. 2016: 192–194).

### Specimens examined

Specimens also examined by Magnes (1997) are denoted by an asterisk (*).

Triblidiaceae

*Triblidiumcaliciiforme*. Austria, Burgenland, Günser Mountains, Oberwart district, Markt Neuhodis, 545 m, 11 Mar 2018, on bark of living *Quercuspetraea*, G. Friebes GJO-0088904. Canada, New Brunswick, Protected Natural Area southeast of Cranberry Lake, 94 m, 11 Jul 2015, on decorticated branch still attached to living *Quercusrubra*, J. M. Karakehian FH-15071105 (FH). Scotland, Mid-Perth (VC 88), south side of Loch Earn, Ardvorlich Woods, 26 Aug 1981, on *Quercus* bark, B. J. Coppins 8659, E-00012551 (E)*. VC 96 Easterness, Aviemore, Torr Alvie SSSI: Bogach carr, 220 m, 23 Sept 2008, on *Salix*, B. J. Coppins and C. J. Ellis [Coppins 22725] E-00905002 (E). United States of America, New York, Ringwood Preserve, 448 m, 1 Aug 2018, on bark of living *Quercusalba*, J. M. Karakehian CUP-18080101 (CUP). —*Huangshaniaverrucosa*. China, Anhwei, Huangshan Mountains, not far from Yun-gu Si, 1 Nov 1980, on bark of *Pinus* sp., O. E. Eriksson 8001101-2a (UME-29336a, isotype)*.

Rhytismataceae, *Pseudographis*

*Pseudographiselatina*. Austria, Styria, Styrian border mountains, Koralpe, Reinischkogelzug Bezirk Deutschlandsberg, 1080 m, 30 Mar 2018, on bark of living *Abiesalba*, G. Friebes GJO-0090016. —*Pseudographispinicola*. Canada, New Brunswick, Charlotte County, Little Lepreau, 28 Sept 2016, on bark of living *Larixlaricina*, J. Tanney FH-NB842 (FH). United States of America, New Hampshire, White Mountain National Forest, Mt. Washington, Tuckerman Ravine Trail, 1058 m, 17 Jun 2018, on bark of fallen log of *Picea* sp., J. M. Karakehian FH-18061706 (FH).

## Discussion

### Recent molecular phylogenetic studies in *Pseudographis* and our results.

Two molecular phylogenetic studies of Leotiomycetes have included isolates of *Pseudographiselatina*. These are Prieto et al. (2018) and Johnston et al. (2019). In their five-gene phylogeny, Prieto et al. (2018) used two genes derived from a *P.elatina* genome, but did not provide an accession number identifying the source of this data. Their phylogeny demonstrated that this *P.elatina* isolate grouped with three other species of Rhytismatales in a well-supported clade and they claimed that their results “confirmed” inclusion of Triblidiales within Leotiomycetes. This finding was incidental to their primary result of identifying the first lichenized lineage within Leotiomycetes. In their 15-gene phylogeny, Johnston et al. (2019) included data from *P.elatina* genome NCBI:txid1695903. Based upon the results of their analysis, they placed Triblidiaceae within Rhytismatales. The results reported by Prieto et al. (2018) and Johnston et al. (2019) support the inclusion of *Pseudographis* within Rhytismatales. However, conclusions regarding the placement of Triblidiaceae within Rhytismatales are speculative. Triblidiales is typified by Triblidiaceae that is, in turn, typified by *Triblidium* and not *Pseudographis*.

Regarding the *P.elatina* genome NCBI:txid1695903, we were initially unable to find any information about the material from which this genome was sequenced. However, we learned that the genome was derived from a culture: CBS 651.97 (Joseph Spatafora pers. com.). The CBS database provides information on the specimen from which the culture was established (Oregon, USA; on bark of living *Pseudotsugamenziesii*; Verkley and Sherwood; October 13, 1996; no. 509), but there is no indication of where this specimen is deposited. We were unable to locate it through online searches.

We included three gene sequences from the *P.elatina* genome (NCBI:txid1695903) in our phylogenetic analysis. Our results indicate that this isolate of *P.elatina* is conspecific with our Austrian isolate (GJO-0090016) (Fig. 3, clade I). Furthermore, our study demonstrates that *Pseudographis* does not cluster with *Triblidium* (Fig. 3, clade A), but with other genera circumscribed within Rhytismataceae.

Magnes (1997) proposed classifying Triblidiaceae in Rhytismatales. The results of our phylogenetic analysis partially support his hypothesis. Triblidiaceae is a monophyletic family composed of *Triblidium* and *Huangshania* (Fig. 3, clade A) that groups within the radiate clade of Rhytismatales (Lantz et al. 2011). *Pseudographis* is not part of Triblidiaceae (Fig. 3, clade F) and groups within the bilateral clade of Rhytismatales (Lantz et al. 2011) (Fig. 3, clade H). According to these results, we have emended the concept of Triblidiaceae and expanded the circumscription of Rhytismataceae to include species of *Pseudographis*. These possess a character novel to the family: ascospore cell walls that produce a strong blue/purple reaction in iodine-based reagents.

### Triblidialean fungi in taxonomic manuals

Triblidialean fungi are not generally treated in modern taxonomic works. Magnes (1997: 16–17) noted that the last most detailed study of *Triblidium* and *Pseudographis* was Rehm’s (1887–1896). Dennis (1981) treated these fungi only superficially. They are not in Korf’s (1973) key to discomycetes and Magnes (1997: 8) claimed that this omission is one of the most important reasons that these fungi have been rarely collected, identified, and deposited in herbaria. Though they are not treated in the lichenological literature, lichenologists may encounter and collect triblidialean fungi as they co-occur in the same habitat as some corticolous and lignicolous lichens.

### The occurrence of triblidialean fungi

Whether a species is common or rare is a question that often arises. Are they rare or are they rarely collected? Regarding triblidialean fungi in Northeastern United States, J.M.K. searched approximately 30 *Picearubens* trees for *P.pinicola* in New Hampshire in June, 2018, and made one collection from an individual tree. In August of that year, in the state of New York, J.M.K. searched approximately 20 *Quercusalba* trees for *T.caliciiforme* and made one collection, as well as one collection of an undescribed *Triblidium* species on a different tree. In May, 2018, J.M.K. visited Newfoundland, Canada to search for a specimen of *Huangshanianovae-fundlandiae* in the type locality and, together with a small group of experienced local botanists, searched approximately two dozen *Pinusstrobus* trees with no result. Our experiences, at least in Northeastern United States, suggest that these fungi are not abundant.

In contrast, it seems that collecting in Europe may be more productive with various species. There are many collections made by Magnes (1997) in Austria and our co-author, G.F., has supplied many fresh collections for this study from some of Magnes’s collecting sites. Observations of other European specimens may be accessed through Baral (2019).

### Potential impact of host tree diseases on distribution of triblidialean fungi

Diseases carried by introduced plants or imported forest products may affect the current and future distribution of triblidialean fungi. Triblidialean fungi are host restricted within woody angiosperms and gymnosperms as far as is known. As an example of narrow host preference we can point to *T.caliciiforme* and our undescribed *Triblidium* species, both of which occur primarily on *Quercus*. Fungal diseases that cause bark decay on living oak trees may impact populations of *Triblidium*. These are referred to as “smooth patch” diseases. They cause the decomposition and sloughing of the rough outer bark, forming regions that are slightly sunken, smooth and lighter in color than surrounding regions. As these regions expand and become confluent on the trunk they become extensive. Smooth patch diseases are caused by species of *Aleurodiscus*, *Dendrothele*, and *Hyphoderma* (all Agaricomycetes, Basidiomycota) (Sinclair and Lyon 2005: 520). Additionally, *Bretziellafagacearum*, causal agent of oak wilt disease, and *Lachnellulawillkommii*, causal agent of European larch canker, are both examples of alien invasive species introduced to North America that cause disease and mortality on triblidialean host tree species.

### On the occurrence of paraphysoids in *Rhytismatales*

The literature is incomplete regarding the occurrence of paraphysoids in Rhytismatales. Nannfeldt (1932: 314) thought that paraphysoids did not occur in *Triblidium* and disagreed with Rehm on the subject. Hawksworth and Sherwood (1982: 264) did not comment on their occurrence in *Triblidium* or *Pseudographis* (Magnes 1997:8). Eriksson (1992: 9) observed paraphysoids in *Huangshania*, and noted them in *Triblidium* and *Pseudographis*. However, he thought they were absent in species of Rhytismatales and therefore declined to place Triblidiaceae within this order, despite similarities in ascus structure.

Magnes (1997: 5, 11–12) noted the occurrence of paraphysoids in Rhytismatales by his own observations of *Triblidiopsispinastri* and considered them a key taxonomic feature of triblidialean fungi. For further documentation, he cited Arx and Müller (1954: 131) who discussed ascomatal development in Hypodermataceae Rehm [= Rhytismataceae]. More supportive, Magnes also cited Gordon (1966: 319–320, 1968: 49) on centrum development in Hypodermataceae that demonstrate the presence of paraphysoids in species of *Lophodermium*. We note that Bellemère (1967) described paraphysoids in the development of ascomata in *Therryafuckelii* (pp. 417–427) and *Colpomajuniperi* (pp. 443–451). Clearly, additional work is needed to document the occurrence of paraphysoids in Rhytismatales.

### On the trophic status of triblidialean fungi and potential endophytism

Little is clearly understood about the trophic mode of triblidialean fungi. Some species, such as *T.caliciiforme* and *P.pinicola*, grow readily on standard culture media (Karakehian, pers. obs.). We presume that ascospores of triblidialean fungi colonize dead woody tissues, especially bark of both living and dead trees, and that they are saprobes. Sherwood (1981: 19) observed that the ascomata of many lignicolous discomycetes inhabit substrata that do not appear to be degraded or affected in any way. This is certainly the case among triblidialean fungi, where ascomata form just below the surface of apparently non-degraded bark and from which they ultimately erupt (see diagram in Rehm 1888: 193, fig. c). We speculate that these fungi exist as a protected, diffuse mycelium growing within bark or the cork cambium, from where they periodically extend hyphae to the surface of the outer bark to produce ascomata. They may also colonize living woody plant tissues and exist as latent saprobes in an endophytic state.

Life histories characterized by alternating endophyte-saprotroph trophic modes are reported among phylogenetically diverse Ascomycota families, for example Dermateaceae (Chen et al. 2016), Mollisiaceae (Kowalski and Kehr 1995; Tanney et al. 2016), Tympanidaceae (Kowalski and Kehr 1992), and Xylariaceae (Okane et al. 2008). Endophytism is common in foliar Rhytismataceae species and is reported in some Rhytismataceae species that produce apothecia exclusively from woody substrates. For example, *Coccomycesstrobi* commonly forms apothecia on dead, self-pruned branches of *Pinusstrobus* and is also reported as a foliar endophyte on the same host (McMullin et al. 2019). Tanney et al. (2018) mentioned the isolation of *Coccomycesirretitus* as a foliar endophyte of *Picearubens*; *C.irretitus* is a bark- and decorticated wood-inhabiting species that is, coincidentally, easily mistaken for *Pseudographis* species in the field. *Tryblidiopsis* species occur on *Picea* and occupy a similar ecological niche as *C.strobi*, occurring as ubiquitous saprotrophs on self-pruned branches but also as endophytes of leaves, cambium, and bark (Kowalski and Kehr 1992; Barklund and Kowalski 1996; Tanney and Seifert 2019). Similarly, *Therrya* species are reported as branch endophytes and implicated in self-pruning of branches in *Pinus* (Kowalski and Kehr 1992; Solheim et al. 2013). *Colpomaquercinum* is one of the most commonly isolated branch endophytes from *Quercusrobur* and is associated with branch pruning (Butin and Kowalski 1983; Kehr and Wulf 1993; Agostinelli et al. 2018). These examples describe a life history strategy that allows the fungus to gain entry into the host (e.g. via foliar infection or branch wounds) and colonize and persist endophytically within cambium and bark until conditions, such as the physiological status of the host substrata, become suitable for more extensive saprotrophic (or weakly parasitic) colonization and subsequent reproduction (Tanney et al. 2016). This latent endophytic colonization likely explains why apothecia of branch-associated Rhytismataceae species are observed soon after branch death, for example caused by shading or physical damage (e.g. lightning; Solheim et al. 2013).

There is currently little evidence of endophytism in triblidialean fungi, although Magnes (1997) conjectured that *Triblidium* species associated with ericaceous shrubs (e.g. *T.carestiae* and *T.hafellneri*) are endophytic based on the rapid formation of apothecia following twig death. That triblidialean life histories are poorly understood is expected given their apparent rarity and the overall paucity of studies investigating branch endophytes and bark fungi associated with triblidialean hosts (e.g. Pinaceae and Ericaceae). The lack of available reference sequences also means that triblidialean species would be unidentifiable in sequence-based studies. Thus, future work should involve ascertaining the extent of host tissue colonization by triblidialean species, for example, isolating from various stem and branch tissues of living hosts exhibiting triblidialean apothecia. Reference sequences generated from this current study will facilitate identification of triblidialean species in future metabarcoding studies and also aid in testing the endophyte hypothesis.

In xeric habitats, wood-inhabiting fungi may not gain the entirety of their nutrition from the degradation of the substrate. It is possible that other sources such as leachates from foliage or epiphytic lichens, insect exudates or bird droppings may come into play. Some of these fungi may also be deriving nutrition from casual associations with algae (Sherwood 1981: 19), as is speculated in *Xerotremamegalospora* (Sherwood and Coppins 1980: 370). From our observations, interaction between algae and triblidialean fungi seems unlikely and it is not known in other Rhytismatales.

### Ascospore morphology of triblidialean fungi

The inclusion of *Triblidium*, *Pseudographis* and *Huangshania* in Rhytismatales introduces ascospore morphologies that were not previously found in the order. We discuss these spore characters because they are novel in the context of non-lichenized, inoperculate discomycetes. These morphologies include: muriform ascospores in *Triblidium* and *Pseudographis*, the virtually opaque dark blue/purple reaction in the ascospore wall in iodine-based reagents in *Pseudographis*, and the large, regularly spaced verrucae and polar cell “plugs” in ascospores of *Huangshaniaverrucosa*.

**Figure 4.**
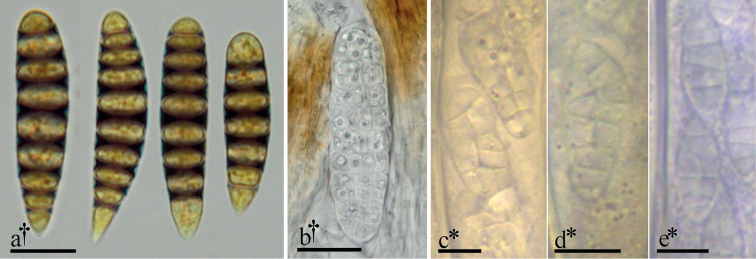
Select examples of ascospore morphologies in Graphidaceae and Leotiomycetes**a** ascospores of *Glyphiscicatricosa* in Lugol’s solution **b** muriform ascospore of *Mellitiosporiumversicolor***c–e** muriform ascospores of *Claussenomyces* spp. within living, immature asci. All microphotographs of cells and tissues mounted in water unless otherwise noted. † = dead, * = living. Scale bars: 10 µm (**a**); 20 µm (**b**); 5 µm = (**c–e**). Specimens photographed: a = J.M.K personal collection; b = U.S.A., Oregon, Horse Rock Ridge, M. A. Sherwood, L. H. Pike & D. Wagner, 21 Mar 1979, FH [s.n.], image courtesy of Farlow Herbarium of Harvard University; **c–e** = L.Q. personal collections.

As outlined in our History, previous classifications have placed *Triblidium* and *Pseudographis* among various groups that now comprise Dothideomycetes, Lecanoromycetes and Leotiomycetes. This has been due in part to an overestimation of the taxonomic significance of their peculiar ascospore characters. Muriform ascospores are more frequently observed in taxa belonging to Lecanoromycetes and Dothideomycetes, and ascospores that display a bluing reaction in iodine-based reagents are commonly observed in Lecanoromycetes, particularly in Graphidaceae (Ostropales) (Fig. 4a). In light of our phylogenetic approach to the classification of this group, we may claim with a greater degree of confidence that these ascospore morphologies have arisen independently within Leotiomycetes.

In order to gain an overview of ascospore morphology within Rhytismatales, we reviewed each genus using a current classification of the order that contains three families: Cudoniaceae, Marthamycetaceae, and Rhytismataceae (Baral in Jaklitsch et al. 2016: 190–194). We generated a spreadsheet of 74 genera, including *Angelina* (Rhytismataceae) (Karakehian et al. 2014), *Triblidium, Huangshania* (both Triblidiaceae) and *Pseudographis* (Rhytismataceae) that were not listed in the published classification under Rhytismatales. We excluded *Tridens*, which has muriform spores and that clearly belongs in Dothideomycetes based on a review of literature and morphological observations of material in FH (Karakehian pers. obs.).

Excluding triblidialean fungi, ascospores of Rhytismatales are generally characterized as hyaline, smooth, filiform, aseptate, enclosed within a gelatinous sheath and supplied with rounded, gelatinous caps at the poles. Many species of commonly encountered genera such as *Rhytisma*, *Coccomyces* and *Colpoma* share this morphology. However, in many genera ellipsoid, cylindric, clavate, fusiform, or hourglass-shaped (bifusoid) ascospores are found. The presence or absence of a gelatinous sheath also varies widely. Ascospore color such as dusky-grays or browns, as well as gelatinous appendages, are reported in a handful of genera (Baral in Jaklitsch et al. 2016: 190–194).

Muriform ascospores, as observed in *Triblidium* and *Pseudographiselatina*, are known in one other genus currently placed in Rhytismatales, *Mellitiosporium* (Fig. 4b). A sequence from a specimen of *M.versicolor*, the type species of the genus according to Dennis (1981: 37 [Addenda and corrigenda]), was included in a gene phylogeny of Rhytismatales by Lantz et al. (2011). *Mellitiosporium* and a handful of other genera were placed in Marthamycetaceae, erected in 2015. This family will be removed from Rhytismatales to its own order, Marthamycetales, based on the results of a study by Johnston et al. (2019). *Triblidium* (Triblidiaceae) and *Pseudographis* (Rhytismataceae) will remain the only genera within Rhytismatales whose members possess muriform spores.

Across Leotiomycetes, muriform ascospores are rare. Nannfeldt (1932: 314) claimed that muriform ascospores were found only in a few species of Helotiales (sensu Nannfeldt) and Korf (1973) observed that their occurrence was so rare and “…almost unheard of… (p. 294)” in discomycetes that “… one should immediately suspect he has a bitunicate ascomycete in hand if they are found (p. 253).” However, Baral in Jaklitsch et al. (2016: 165–166) noted that, in regards to Dermateaceae (Helotiales), ascospores may become “septate to muriform.” We reviewed the work of Baral in Jaklitsch et al. (2016: 157–205), Korf (1973), Sherwood (1981: 20–21) and Dennis (1981) for the presence of muriform ascospores in Leotiomycetes. We found that, in addition to *Mellitiosporium* and Baral’s observation regarding Dermateaceae (probably referring to *Pezicula*), that muriform ascospores are also observed in members of *Claussenomyces* (Tympanidaceae) (Fig. 4c–e) and in *Waltoniapinicola* (Helotiales*incertae sedis*).

The taxonomic significance of ascospore septation should be considered with caution. Although spore septation has been used as a character in fungal classification it is unreliable as a single trait. Both muriform and transverse-septate ascospores are observed among species of the same genus in Graphidaceae (Staiger et al. 2006: 769–771). This is the case in other groups of lichens (Lumbsch et al. 1997; Nelson et al. 2014; Luangsuphabool et al. 2018) as well as in non-lichenized taxa (Mugambi and Huhndorf 2009). In the triblidialean fungi, among species of *Pseudographis* we observe muriform spores in *P.elatina* and strictly transverse-septate spores in *P.pinicola*.

Muriform ascospores may have adaptive significance in harsh terrestrial ecosystems where suspended, exposed bark and wood are potential substrata for colonization. All non-lichenized muriform-spored discomycetes occur in this habitat (Sherwood 1981: 26). Calhim et al. (2018) analyzed spore morphology in relation to trophic modes and substrate associations. Their results indicated that in regards to evolutionary drivers of spore morphology, deposition of undamaged spores on specific substrates is more strongly selected for than is spore dispersal. Large, muriform ascospores of the type produced by *Triblidium* species represent an evolutionary approach to the mitigation of damage that may occur during spore transport and deposition. Gregory (1961: 82) stated that deposition by wind impaction is more efficient for large spores and Sherwood (1981: 25) noted that longitudinal and transverse septa increase structural stability, eliminating the need for a very thick wall.

Muriform spores may also present advantages in the efficient colonization of substratum. The larger number of cells in muriform ascospores increase the chances of successful colonization even if some cells are damaged in transport or deposition (Sherwood 1981: 26). Furthermore, rapid colonization may be achieved by the simultaneous germination of many cells. The germ tubes form an advancing perimeter of hyphae around the deposited spore (Fig. 1j). Finally, if not every cell germinates at once, and if the ascospore happens to be dislodged during germination or if conditions turn adverse before the fungus becomes established, then the ungerminated cells may represent additional chances for successful colonization.

The intense blue/purple reaction of the ascospores of *Pseudographis* species in iodine reagents is unique within Leotiomycetes. There are no other species within Rhytismatales that share this character. In Leotiomycetes, a tepid blue or blue-green reaction is reported in spores of *Strossmayeria* and in the related species *Durellaconnivens* (both Helotiales, *Strossmayeria* lineage) (Baral in Jaklitsch et al. 2016: 176–177).

In the ascospores of *Strossmayeria* species, the reaction appears to be erratic. It is not entirely clear to us if it occurs in the ascospore wall, gel sheath, or both. Iturriaga and Korf (1990: 383) stated that the blue reaction of the ascospores is a “rare phenomenon in Ascomycetes”. It occurs in both ascospores and ectal excipulum. It is generally lighter in the ascospores and is highly variable in that it may be fleeting, long-lasting or not occurring for hours. The reaction was reported to be stronger in asci containing mature ascospores (possibly due to agglomeration), and was particularly strong in the ascospore and gel layer of *S.jamaicensis* (p. 433). We attempted to observe the blue reaction in material collected in 2016 from Mozambique. This failed even with KOH pretreatment. We then tried with dried material from FH that was 30 years old and observed an extremely faint reaction in only a few asci that contained mature-appearing ascospores.

Though the ascospore iodine reaction is equally intense in *Pseudographis* species and many species of lichenized Ascomycota in Graphidaceae (Ostropales, Lecanoromycetes), the reaction is localized differently in the spore walls of the two groups. In *Pseudographis*, the reaction is entirely uniform across the ascospore surface. In undiluted iodine reagent mounts the coloring may be so opaque that the septa are obscured even at the highest illumination settings in transmitted light microscopy. The reacting material presumably occurs within the cell wall or in some coating on the very surface of the ascospore (Fig. 2k, m, p, r). In comparison, in ascospores of Graphidaceae species, the spore walls are laminate, with a non-reactive outer spore wall and a reactive inner wall that stains dark blue/purple black. This reactive material surrounds the inner cells, and the lumina and cell walls of these remain clearly observable in light microscopy even in undiluted iodine reagents (Fig. 4a).

The dark-blue/purple reaction in *Pseudographis* ascospores leads to questions regarding the composition and structure of the reacting substance, as well as its biological role. Because the quality of the staining reaction appears to be analogous to what is observed in the cell walls and surface ornamentations of basidiospores in genera such as *Lentinellus*, *Russula* and *Amanita* in Agaricomycetes (Basidiomycota), we began to address these questions by searching for literature on amyloid reactions in Basidiomycota.

Dodd and McCracken (1972) reported on the molecular structure and biological role of polysaccharides in selected Agaricomycetes genera that possess amyloid basidiospores and basidiome tissues. The authors proposed that the starch on the surface of basidiospores may act as a permeability barrier to maintain dormancy. A thin layer of amylose molecules (one of two components of starch) may serve as an oxygen barrier, slowing metabolism and thus conserving nutrients within the spore. As the amylose molecules are cold-water soluble, when environmental conditions are humid the layer will dissolve to some threshold degree that allows oxygen to diffuse into the spore. Thus, basidiospores remain dormant and viable for a period until environmental conditions are optimal for growth.

Are *Pseudographis* ascospores enveloped in a starch-based, degradable film? Basic research in the chemistry and physical properties of these films as water and oxygen barriers in biodegradable food packaging may offer some insights (Rindlav-Westling et al. 1998; Stading et al. 2001; Forssell et al. 2002). Starch-based films are hydrophilic and their water permeabilities are affected by humidity. Water sorption occurs naturally in these films. A proposed mechanism for increased oxygen and water permeability in starch-based films is that as water is taken up, the network of crystalized strands in the film becomes heterogeneous. The film swells as humidity increases and pores of various sizes form. Oxygen permeability increases dramatically at approximately 60–70% ambient humidity (Stading et al. 2001: 209, 212). This model of starch-film modification and permeability is alternative to the one proposed by Dodd and McCracken (1972), where some component of starch is removed. Ascospores enveloped in a hygroscopic film is a conceivable adaptation to the xeric substrates and habitats characteristic of triblidialean species.

To conclude our Discussion, we will discuss ascospore wall sculpturing and the terminal cell structures observed in *Huangshaniaverrucosa* (Fig. 1r–t). There are no other species within Rhytismatales with ascospore surface sculpturing. This feature is rare within the related order Helotiales (Leotiomycetes) with ascospores of *Drepanopezizaverrucispora* with acute spines and *Mollisiadextrinospora* ornamented by fine verrucae, for example.

The evolutionary and ecological significance of spore ornamentation is only beginning to be addressed by recent research. However, Pringle et al. (2015: 214) stated that to their knowledge, there is no research on the evolutionary origin of spore ornamentation, or even whether smooth spores represent a loss from an ornamented ancestral state. Both Halbwachs et al. (2015) and Calhim et al. (2018) demonstrated that spore ornamentation occurred more frequently in ectomycorrhizal genera than in saprobic ones. Halbwachs et al. (2015 p. 198), citing Lilleskov and Bruns (2005), speculated that spore ornamentation may facilitate adhesion to invertebrate exoskeletons in the delivery of spores of ectomycorrhizal species deep enough into the soil to germinate near fine root tips of host species.

How then might spore ornamentation be advantageous for saprobic species that colonize above-ground substrates? In light of the system described by Reynolds (2013), wherein passively dispersed organisms travel only so far as to the nearest unoccupied location from the parent in order to escape depleted resources, but not so far away as to leave behind a resource pool that is reliable and predictable, we may consider that ascospores of *H.verrucosa* do not travel far from parent ascomata. This implies that bark is the preferred substratum on which ascospores are to be deposited due to impaction. We may then speculate that the roughened ascospore surface may be an adaptation to facilitate adhesion to bark or other woody tissues. The increased adhesive quality of the ascospores might prevent them from being dislodged by wash-out or wind during deposition as well as subsequent germination and colonization phases.

In *H.verrucosa* there is an extension of the sporoplast or an otherwise differentiated structure in the terminal cells of ascospores (Eriksson 1992: 4). We also observed this feature (Fig. 1p, r). Magnes (1997: 77) described these as round appendages approximately 2 μm wide. Eriksson (1992: 4, fig. 3, 7) described them as “firm, subspherical to conical, plug-like appendages.” They do not differentially stain in Congo red or in cotton blue in lactophenol. Without data from ascospore germination experiments it is unknown if these structures may act as germ pores. Excluding triblidalean fungi, ascospores in Rhytismatales are predominantly thin-walled, and we are not aware of the occurrence of germ pores in any taxon in the order.

## Conclusion

The history of Triblidiaceae is one among many cases in systematic mycology of the challenges present in the classification of fungi that result from the use of seemingly distinctive morphological characters, such as ascospore morphology, that are unreliable when tested using molecular phylogenetic methods. Our research supports Magnes’s hypothesis of the relationship of *Triblidium*, *Huangshania* and *Pseudographis* within Rhytismatales. However, we have restricted his concept of Triblidiaceae to circumscribe *Triblidium* and *Huangshania* and we have expanded the circumscription of Rhytismataceae to include *Pseudographis*. Our results have allowed us to investigate ecosystem pressures that have selected for these distinctive ascospore morphologies from a phylogenetically informed perspective. Discomycetes inhabiting desiccated standing or suspended dead wood or bark substrata face the same rigors as epiphytic lichens and lichenicolous fungi. These have convergently evolved many ascomatal features that are unique to this habitat and differ from those discomycetes that occur in mesic habitats. These characters include dark, stromatic excipular tissues that close over the hymenium in dry conditions, pigmented epithecia (exudates), and muriform ascospores (Sherwood 1981: 15–16). Molecular phylogenetic methods and more comprehensive taxon sampling may produce more robust hypotheses of evolutionary relationships under a phylogenetic species concept (Taylor et al. 2000). This approach, combined with analysis of morphology in the context of the severe constraints imposed by the habitat, may aid in elucidating testable questions in the biology and ecology of these organisms.

## Supplementary Material

XML Treatment for Triblidiaceae
